# Global Health Training—One Way Street?

**DOI:** 10.4269/ajtmh.2011.10-0694a

**Published:** 2011-03-04

**Authors:** J. Jaime Miranda, Patricia J. Garcia, Andres G. Lescano, Eduardo Gotuzzo, Hector H. Garcia

**Affiliations:** School of Medicine; CRONICAS - Centro de Excelencia en Enfermedades Crónicas; Universidad Peruana Cayetano Heredia; Miraflores; Lima, Perú; E-mail: Jaime.Miranda@upch.pe; School of Public Health; Universidad Peruana Cayetano Heredia; San Martin de Porres; Lima, Perú; E-mail: patricia.garcia@upch.pe; Department of Parasitology and; Public Health Training Program; U.S. Naval Medical Research Unit 6; Lima, Perú; School of Public Health; Universidad Peruana Cayetano Heredia; San Martin de Porres; Lima, Perú; E-mail: willy.lescano@med.navy.mil; Instituto de Medicina Tropical “Alexander Von Humboldt”; Universidad Peruana Cayetano Heredia; Lima, Perú; E-mail: eduardo.gotuzzo@upch.pe; Department of Microbiology; School of Sciences; Center for Global Health – Tumbes; Universidad Peruana Cayetano Heredia; Lima, Perú; E-mail: hgarcia@jhsph.edu

Dear Sir:

In the last issue of the *Journal*, Crump and Sugarman, on behalf of the Working Group on Ethics Guidelines for Global Health Training,^1^ propose a set of ethics and best practice guidelines for training experiences in global health. The manuscript acknowledges the issues of reciprocity and long-term partnerships between developed and developing country partners. However, global health training, as the manuscript reflects, is still focused on training students from rich countries (paying high tuition fees to their institutions), and not quite contributing to leverage the underlying disparities.

We want to comment on the important issue of bi-directionality, introduced early on page 1179 (“*Although the guidelines are predominantly focused on ethical issues for programs sending trainees from wealthier to less wealthy settings, many of the principals also apply to bi-directional trainee exchanges*”) and not further elaborated in the paper. As health researchers living and working in a developing—less wealthy, less developed, under-resourced, or poorer—country, we have to disagree with such a limited conception. If the goal is to maximize benefit for every party involved—assumedly this is precisely why it is called global health—then these guidelines and their potential impact will probably be very limited. The proposed guidelines basically disregard the big challenges (mainly economical) and great potential advantages of bi-directional flow of trainees. Furthermore, scarce consideration has been given to South-South training exchanges, which could be more culturally appropriate. We hope this comment can contribute to give more consideration and support to the North-South, South-North, and South-South flows of trainees, in the settings of real “global” health.
